# Hydrodynamics-Based Functional Forms of Activity Metabolism: A Case for the Power-Law Polynomial Function in Animal Swimming Energetics

**DOI:** 10.1371/journal.pone.0004852

**Published:** 2009-03-31

**Authors:** Anthony Papadopoulos

**Affiliations:** Department of Biological Sciences, Texas Tech University, Lubbock, Texas, United States of America; Universidad Europea de Madrid, Spain

## Abstract

The first-degree power-law polynomial function is frequently used to describe activity metabolism for steady swimming animals. This function has been used in hydrodynamics-based metabolic studies to evaluate important parameters of energetic costs, such as the standard metabolic rate and the drag power indices. In theory, however, the power-law polynomial function of any degree greater than one can be used to describe activity metabolism for steady swimming animals. In fact, activity metabolism has been described by the conventional exponential function and the cubic polynomial function, although only the power-law polynomial function models drag power since it conforms to hydrodynamic laws. Consequently, the first-degree power-law polynomial function yields incorrect parameter values of energetic costs if activity metabolism is governed by the power-law polynomial function of any degree greater than one. This issue is important in bioenergetics because correct comparisons of energetic costs among different steady swimming animals cannot be made unless the degree of the power-law polynomial function derives from activity metabolism. In other words, a hydrodynamics-based functional form of activity metabolism is a power-law polynomial function of any degree greater than or equal to one. Therefore, the degree of the power-law polynomial function should be treated as a parameter, not as a constant. This new treatment not only conforms to hydrodynamic laws, but also ensures correct comparisons of energetic costs among different steady swimming animals. Furthermore, the exponential power-law function, which is a new hydrodynamics-based functional form of activity metabolism, is a special case of the power-law polynomial function. Hence, the link between the hydrodynamics of steady swimming and the exponential-based metabolic model is defined.

## Introduction

Activity metabolism represents the relationship between metabolic rate and steady speed. Any functional form that is an interpolant of activity metabolism can be used to describe activity metabolism. For example, the conventional exponential function and the cubic polynomial function have been used to describe activity metabolism for steady swimming animals [1]–[6]. In theory, activity metabolism can also be described by the *n*th-degree power-law polynomial function, that is, a fractional polynomial function of the form 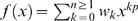, where power *p* (≠0) and coefficients 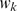 are real-valued parameters [7]. (Note that if *kp* are exclusively natural numbers, then *f* (*x*) is a conventional polynomial function of *np* degree.) In particular, the first-degree power-law polynomial function is frequently used to describe activity metabolism for steady swimming animals to evaluate important parameters of energetic costs, such as the standard metabolic rate and the drag power indices [5], [8]–[10]. Although many functional forms can be used to describe activity metabolism, only the power-law polynomial function models drag power for steady swimming animals [11]. In fact, the power-law polynomial function can be characterized as a power series in the Reynolds number, which is used to describe the Oseen drag coefficient to evaluate drag on a sphere [12]–[14]. Thus, only the power-law polynomial function conforms to hydrodynamic laws [5], [8], [11], [15], [16]; it is because of this important property that the first-degree power-law polynomial function is the standard functional form used in all hydrodynamics-based metabolic studies. Nonetheless, the first-degree power-law polynomial function describes only one of many hydrodynamics-based functional forms of activity metabolism. Consequently, if activity metabolism is governed by the power-law polynomial function of any degree greater than one, then the standard functional form yields incorrect parameter values, leading to incorrect comparisons of energetic costs among different steady swimming animals. Therefore, the objective of this manuscript is to resolve the issue by first deriving the power-law polynomial function, in which the degree is unconstrained, and then showing that this function always describes the correct functional form governing activity metabolism. Moreover, I show that the power-law polynomial function describes many hydrodynamics-based functional forms of activity metabolism; one such functional form is the exponential power-law function. I thus provide a new link between the hydrodynamics of steady swimming and the exponential-based metabolic model. Finally, I show that if different hydrodynamics-based functional forms share the same activity metabolism, then different degrees of the power-law polynomial function yield different parameter values of energetic costs, implying that only with the power-law polynomial function, in which the degree derives from activity metabolism, can one obtain the correct values of the standard metabolic rate and the drag power indices.

### The power-law polynomial function

The first-degree power-law polynomial function, which is the standard functional form used in hydrodynamics-based metabolic studies, is [5], [8]–[10]:
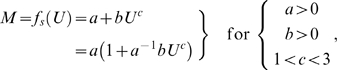
(1)and its natural logarithm-transformed linear form is:
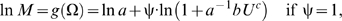
(2)where dependent variable *M* is the total metabolic rate, independent variable *U* is the steady swimming speed, variable Ω (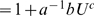) is the relative energetic cost of drag, parameter *a* is the standard metabolic rate, parameters *b* and *c* are drag power indices, and parameter ψ (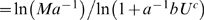) is the metabolic-to-drag power conversion index, which depends on physiological factors similar to factors affecting *b* (e.g., capillary and mitochondrial densities) and physical factors similar to factors affecting *c* (e.g., pressure and viscous drag forces). Note that the variable 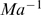 (>1) is the relative metabolic cost of steady swimming [17], which is equal to Ω only if ψ is equal to 1. The parameters *a*, *b*, *c*, and ψ represent energetic costs: *a* is the minimum metabolic rate needed to sustain physiological maintenance [2], [6], [18]; *b* and *c* are inversely related to the swimming capacity and swimming efficiency, respectively [5], [9], [15], [19]; and ψ is inversely related to the power conversion efficiency ( = drag power over metabolic power). For convenience, it is assumed that measurement error, that is, stochastic and systematic variation in 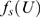 irrelevant to the hydrodynamics, is negligible; this implies that *a*, *b*, *c*, and ψ are realized values, not estimates.

Equation (2) is the log-linear form of equation (1). Thus, like any log-linear function, equation (2) has an intercept ( = ln *a*) and a slope ( = ψ), which is constrained to 1 to satisfy equation (1). The assumption that follows from this constraint is that all of the metabolic power ( = *M*−*a*) required to overcome hydrodynamic drag converts into drag power (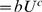) [11], even though a power conversion efficiency of 1 is unattainable [20], [21]. For steady swimming fish, equation (1) and direct hydromechanical models yield similar estimates of drag power [11], suggesting that ψ is close to 1.

Equation (1) tacitly assumes a constant ψ of 1 and thus does not take into account the differences in the power conversion efficiency, which is usually different for individuals within species and almost always different for individuals among species. The Second Law of Thermodynamics explicitly states that the power conversion efficiency is always less (never greater) than 1 [21]. Hence, drag power is always less than metabolic power; this is because not all of the metabolic power required to overcome hydrodynamic drag converts into drag power, some is lost as heat due to physiological and physical factors [20]; then to compensate for heat loss, metabolic power must be greater than drag power. As a result, the metabolic-to-drag power conversion index (ψ) is always greater (never less) than 1. Therefore, to ensure correct comparisons of energetic costs among different steady swimming animals, ψ must be treated as a parameter, not as a constant.

If ψ is treated as a parameter, then the power-law polynomial function derives from the antilogarithm-transformed curvilinear form of equation (2) for ψ greater than or equal to 1:
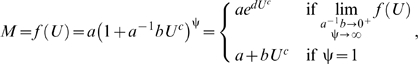
(3)where the exponential power-law function is:
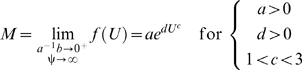
(4)and parameter *d* is equal to 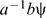 (Methods, equations 10–13). Thus, *d* represents a three-way interaction between *a*, *b*, and ψ; if the cost of physiological maintenance is much greater than the cost of swimming, that is, if the value of 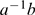 is much less than 1, and if the cost of power conversion is very high, that is, if the value of ψ is much greater than 1, then equation (3) converges to equation (4) because the limit of *f* (*U*) as 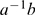 approaches 0 and ψ approaches ∞ defines the exponential power-law function (Methods, equations 10–13). Naturally, if ψ is equal to 1, then equation (3) is equivalent to equation (1). Note that, like equation (1), equation (4) is a hydrodynamics-based model because it is a special case of equation (3); also, if *c* equals 1, which does not conform to hydrodynamic laws, then equation (4) is the conventional exponential function. Furthermore, like equation (1), equation (3) derives from hydrodynamics, from which the compound parameter *c*ψ is inversely related to the overall energetic efficiency (Methods, equations 14–17).

The four parameters (*a*, *b*, *c*, and ψ) in equation (3) can be easily evaluated by maximum likelihood parameter estimation; for best results, the following constraints should be imposed: *a*>0, *b*>0, *c*>1, and ψ≥1; and lognormal error should be assumed since equation (2) is a log-linear function.

### Hydrodynamics-based functional forms of activity metabolism

Equation (3) links the total metabolic rate (*M*) to a power-law polynomial function and thus describes many hydrodynamics-based functional forms of *f* (*U*); this can be shown by expanding equation (3) using the Maclaurin series [22]. Thus, the following expansion is equivalent to equation (3) (Methods, equations 18–21):
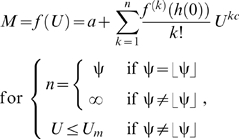
(5)where the floor function 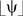 is the highest integer less than or equal to ψ, the parameter 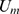 (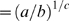) is the maximum sustained *U* at which 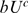 equals *a*, the summation term 
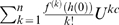
 represents drag power, *h*(*U*) is equal to 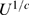, and 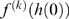 is a differential sequence of coefficients that contain the parameters *a*, *b*, and ψ (Methods, equation 20). It is important to note that if ψ is not an integer, then equation (5) converges to an unspecified *n*th (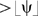) degree power-law polynomial because ∞ is not a number; for this case, the value of *n* depends on the precision with which the convergence is calculated. Conversely, if ψ is an integer, then equation (5) converges to a specified *n*th (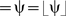) degree power-law polynomial. Also, if 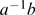 approaches 0 and ψ approaches ∞, then equation (5) equals:
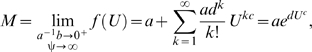
(6)where the limit 
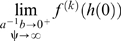
 is equal to 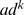. It is apparent from equation (6) that the exponential power-law function is a power-law polynomial function, since ψ converges to a value much greater than 1, and thus conforms to hydrodynamic laws; also, the conventional exponential function ( = equation 6 for *c* = 1) is simply a conventional polynomial function and thus, like the cubic polynomial function, does not conform to hydrodynamic laws. Investigators who use the conventional exponential function tacitly assume *c* is 1, even though they provide no theoretical support for this assumption. As a result, constraining *c* to 1 should not be imposed.

**Figure 1:**
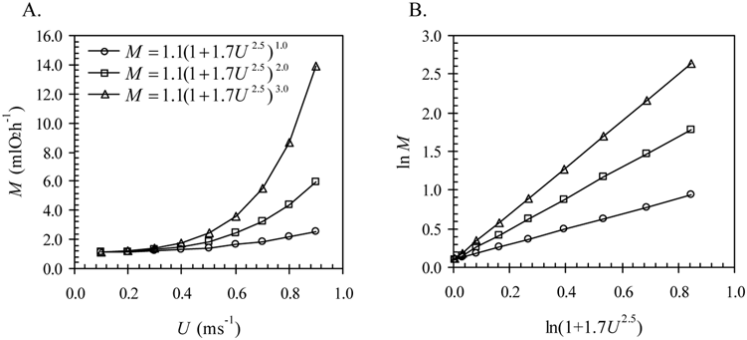
The data represent the curvilinear and log-linear forms of activity metabolism. A. The data are described by the power-law polynomial function (equation 3). All three curves represent different hydrodynamics-based functional forms of *f* (*U*), even though parameters *a*, *b*, and *c* are shared. Note that only the values of ψ are different. Circles, squares, and triangles are characterized by the first-degree power-law polynomial function (ψ = 1; equation 1), the second-degree power-law polynomial function (ψ = 2), and the third-degree power-law polynomial function (ψ = 3), respectively. B. The data correspond to the log-linear form of *f* (*U*), or correspond to *g*(Ω) (equation 2).

According to equation (5), the degree of the power-law polynomial function (or the value of *n*, which depends on the value of ψ) corresponds to the functional form of *f* (*U*); for example, the first-degree power-law polynomial function (*n* = 1 if ψ = 1), the second-degree power-law polynomial function (*n* = 2 if ψ = 2), and the third-degree power-law polynomial function (*n* = 3 if ψ = 3) are three different hydrodynamics-based functional forms of *f* (*U*) (Figure 1A). The power-law polynomial function is not only based on hydrodynamic principles, but also represents the generalized functional form governing *f* (*U*). Equation (3) is complete because any value of ψ does not modify the functional form of equation (2)—that is, the functional form of equation (2) remains log-linear for any value of ψ (Figure 1B); this implies that all hydrodynamics-based functional forms account for the same amount of systematic variation in *f* (*U*) relevant to the hydrodynamics. Thus, fitting different hydrodynamics-based functional forms of *f* (*U*) to the same *f* (*U*) results in similar correlation coefficients and in similar optimum swimming speeds (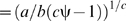), which are useful for comparing transport costs among different steady swimming animals [23]. This important result must hold because the logarithm of metabolic power ( = ln(*M*−*a*)) is a linear function of the logarithm of drag power [8], [10], [15], [16].

### Differences in the parameter values among different degrees of the power-law polynomial function

The parameters *a*, *b*, and *c* are extensively used in hydrodynamics-based metabolic studies because these parameters have useful hydrodynamic and metabolic interpretations [5], [8], [9], [15], [19], [24], [25]. For instance, the standard metabolic rate (*a*) is used in numerous contexts, such as in growth [25]–[27], in morphology [25], [28], and in swimming performance [25], [29]. The drag power indices (*b* and *c*) are also used in numerous contexts, especially in regard to hydrodynamics; for example, *b* and *c* are used to calculate the dimensionless drag indices derived from the function describing the relationship between the drag power coefficient and the Reynolds number (see Appendix 1 in Papadopoulos [5]), and thus are useful for comparing drag power among different steady swimming animals [30]. In particular, because *c* is directly related to the drag exerted by the water on the animal's body [5], [9], [19], it can be used to assess the relationship between body shape and swimming efficiency [24], [25]. Indeed, the parameters *a*, *b*, and *c* have broad ecological and evolutionary significance [25]. Yet, fitting different hydrodynamics-based functional forms of *f* (*U*) to the same *f* (*U*) results in different parameter values; equation (1) yields incorrect values of *a*, *b*, and *c* if equation (3) for ψ greater than 1 governs *f* (*U*); this is an inherent bias of equation (1), which is the standard equation used in all hydrodynamics-based metabolic studies.

I show that fitting different hydrodynamics-based functional forms of *f* (*U*) to the same *f* (*U*) results in different values of *a*, *b*, and *c*. Note that a hydrodynamics-based functional form of *f* (*U*) is a power-law polynomial function of any degree greater than or equal to 1; the degree of the power-law polynomial function corresponds to the value of *n*, which ultimately depends on the value of ψ.

Only the standard metabolic rate (*a*) is evaluated by extrapolating to zero *U*; and because the extrapolant of *f* (*U*) solely depends on the interpolant of *f* (*U*), the value of *a*, like the values of *b* and *c*, depends on the functional form used to interpolate *f* (*U*). In other words, different values of the metabolic-to-drag power conversion index (ψ) result in different values of *a*, *b*, and *c*; this can be shown mathematically by applying composite function operators to *f* (*U*).

**Figure 2:**
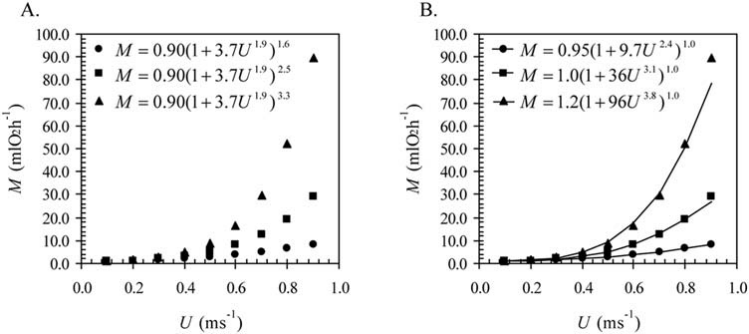
The data represent activity metabolism, which is described by the power-law polynomial function (equation 3). A. Hypothetical representation of actual observed data, where circles represent *f* (*U*) for individual 1 (*a* = 0.90; *b*/*a* = 3.7; *c* = 1.9; ψ = 1.6), squares represent *f* (*U*) for individual 2 (*a* = 0.90; *b*/*a* = 3.7; *c* = 1.9; ψ = 2.5), and triangles represent *f* (*U*) for individual 3 (*a* = 0.90; *b*/*a* = 3.7; *c* = 1.9; ψ = 3.3). Note that only the values of ψ are different. B. The curve-fit of equation (1), in which ψ = 1.0, to the actual observed data. Note that the parameters values of *a*, *b*, and *c* from equation (1) are incorrect (see Table 1 for their deviations).

If *f* (*U*) is governed by equation (3) for ψ equal to α, but equation (3) for ψ equal to β (≠α) is used to interpolate *f* (*U*), then 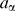 (α-specific value of *a*) and 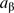 (β-specific value of *a*) can be evaluated using the following first derivative of the composite function:
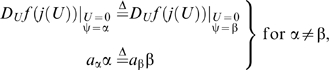
(7)where *j*(*U*) is equal to 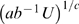; similarly, *b* and *c* can be evaluated using the following first derivative of the composite functions:
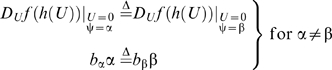
(8)and
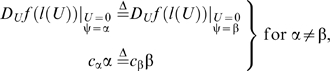
(9)where *h*(*U*) and *l*(*U*) are equal to 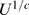 and 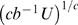, respectively. Equations (7)–(9) are conditional, implying that equality is guaranteed only if different hydrodynamics-based functional forms of *f* (*U*) share the same *f* (*U*); otherwise, this analysis is inconclusive. Since α is not equal to β, but 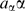, 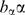, and 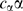 are equal to 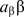, 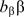, and 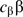, respectively, it must follow that 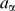, 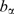, and 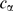 are not equal to 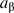, 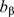, and 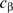, respectively. Because *f* (*U*) is governed by equation (3) for ψ equal to α, 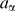, 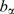, and 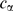 are the only correct parameter values. This is a very important result: there is only one correct functional form governing *f* (*U*); and only with equation (3), in which parameter ψ is a consequence of *f* (*U*), can one obtain the correct parameter values. Note that because only *a* is the value of *f* (*U*) at *U* equal to 0, 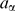 is approximately equal to 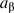 if the values of Ω are small enough such that different values of ψ result in similar values of *a* for the same *f* (*U*); this rare condition can only occur when *a* is much greater than *b* and most of the measured values of *U* are less than 1. Also, if α is less than β, then it must follow that 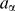, 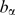, and 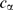 are greater than 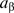, 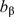, and 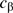, respectively. Thus, if equation (3) for ψ greater than 1 governs *f* (*U*) (see Figure 2A), but equation (1), that is, equation (3) for ψ equal to 1, is used to interpolate *f* (*U*) (see Figure 2B), then the values of *a*, *b*, and *c* derived from equation (1) are not only incorrect, but also greater than the correct values of *a*, *b*, and *c* (Table 1).

**Table 1:** A comparison of the parameters of energetic costs among different individuals.

Individual 1 (•)	*a*	*b*	*c*	ψ
actual values from *f* (*U*)	0.90	3.3	1.9	1.6
incorrect values from 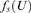	0.95	9.2	2.4	1.0
deviation from *f* (*U*)	0.050	5.9	0.50	−0.60

Figures 2A and 2B illustrate the importance of using only equation (3) to interpolate *f* (*U*). In Figure 2A, all three individuals have the same values of *a*, *b*, and *c* (Table 1). Yet, fitting equation (1) to *f* (*U*) results in all three individuals having different values of *a*, *b*, and *c* (Figure 2B; Table 1). One would then conclude from equation (1) that the cost of physiological maintenance (*a*) and the costs of swimming (*b* and *c*) among the three individuals are different, but, in fact, they are not. It is due to the different values of only ψ that the three curves in Figures 2A and 2B are different. In other words, it is only the differences in the power conversion efficiency that makes the three curves appear different. Equation (3) takes into account the differences in ψ, whereas equation (1) tacitly assumes that there are no differences in ψ—that is, equation (1) tacitly assumes ψ is equal to 1, but ψ is really equal to 1.6, 2.5, and 3.3 for individual 1, 2, and 3, respectively (Table 1). Making the assumption that ψ is constant is clearly flawed and thus leads to incorrect comparisons of energetic costs among different steady swimming animals. Therefore, equation (3) must, by definition, overrule any special-case function (e.g., equations 1 and 4) because it is a generalized hydrodynamics-based model; any value of ψ (≥1) is justified if it is a consequence of *f* (*U*).

How would one interpret the association between the energetics and the hydrodynamics of steady swimming from the observed data in Figure 2A? First, the power conversion efficiency decreases as the values of ψ increase, from 1.6 to 3.3, implying that some of the metabolic power is transformed into heat; and thus, to compensate for the heat loss, a supplement of metabolic power is required to overcome hydrodynamic drag. Consequently, if the incorrect model (equation 1) is used to interpolate *f* (*U*), then the heat loss is completely converted into drag power, which is physiologically impossible [20]. Second, because the cost of physiological maintenance and the costs of swimming are the same for all three individuals, but the cost of power conversion is different, implies that only the metabolic response due to the conversion of biochemical energy to mechanical power of the muscles is different among the three individuals. Although this is an interesting hypothetical case, the difference in only ψ among the three individuals is most likely due to the difference in ventilatory capacity as opposed to the difference in respiratory capacity, since the values of *b* are the same.

## Discussion

For over 30 years, the first-degree power-law polynomial function (equation 1) has been used to describe *f* (*U*) for steady swimming animals, especially for fish [5], [8]–[10], [15], [19], [24], [25], [31]–[33]. Concurrently, the conventional exponential function ( = equation 4 for *c* = 1) has also been used to describe *f* (*U*) [1], [2], [6], [15], [25], [31]–[33]. Some argue that equation (1) is more appropriate than the conventional exponential function because only equation (1) is based on hydrodynamic principles [9], [11]. Yet others argue that the conventional exponential function is a more robust predictor of *f* (*U*) than equation (1) because the conventional exponential function has two, as opposed to three, parameters [15], [33]. In fact, both arguments are disputable: equation (1) and equation (4), which is a generalization of the conventional exponential function, are hydrodynamics-based models because they represent special cases of equation (3); and, with or without measurement error, equations (1) and (4) predict *f* (*U*) with a similar level of statistical robustness because both functions have the same number of parameters, that is, three.

Two methods can be used to formulate equation (3): in the first method, equation (1) is factored into two multiplicative parts, *a* and Ω, and the natural logarithm of the factorization is calculated, thus exposing the model as a log-linear function (equation 2), in which the intercept ( = ln *a*) and the slope ( = ψ) are defined—variable Ω and parameters *a* and ψ have important biological interpretations; in the second method, *f* (*U*) is derived from hydrodynamics (Methods, equations 14–17). In both methods, ψ is assumed to be constant (that is, 1), thus characterizing *f* (*U*) as a first-degree power-law polynomial function, making *f* (*U*) incomplete; however, by simply allowing ψ to be a parameter, *f* (*U*) becomes complete (equations 3 and 5; Figure 1A). As result, a high degree power-law polynomial function (that is, ψ≫1) converges to equation (4) as 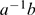 approaches 0 (equation 6).

Like all hydrodynamics-based functional forms of *f* (*U*), the conditions that satisfy equation (4) have a biological interpretation: the standard metabolic rate (*a*) must be much greater than the drag power index (*b*), and the metabolic-to-drag power conversion index (ψ) must be much greater than 1. Note that if ψ is close or equal to 1 and 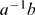 is much less than 1, then equation (3) converges to *a* because the limit of *f* (*U*) as 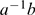 approaches 0 and ψ approaches 1 equals *a*, which is nonsensical because *a* is, by definition, a parameter, not a variable. Thus, for equation (3) to make sense, ψ must be much greater than 1 only if 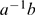 is much less than 1. Remarkably, this three-way interaction is also fundamental to the definition of equation (4) (Methods, equations 10–13).

In conclusion, equation (3) describes many hydrodynamics-based functional forms of *f* (*U*) because it is characterized as a power-law polynomial function (equation 5). Different hydrodynamics-based functional forms, or different degrees of the power-law polynomial function yield different values of *a*, *b*, and *c* for the same *f* (*U*) (equations 7–9; Figures 2A and 2B; Table 1). Thus, it is important that equation (3), not equation (1), is used because equation (1) describes only one of many hydrodynamics-based functional forms of *f* (*U*); equation (1) can only yield the correct values of *a*, *b*, and *c* if ψ is very close or equal to 1, either of which is theoretically justified for only fish [11]. Yet, because hydrodynamic laws permit ψ to take any value greater than or equal to 1 (Figure 1B), ψ should always be treated as a parameter, not as a constant.

## Methods

### Derivation of the exponential power-law function from *f* (*U*)

The following derivation of the exponential power-law function (equation 4) is an adaptation of the definition of the conventional exponential function 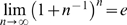 first proposed by Jacob Bernoulli in 1683 and then generalized to 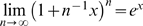 by Leonhard Euler in 1748 (see Example 8 on page 392 in Finney and Thomas [22]). Start with the natural logarithm of equation (3):
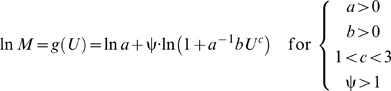
(10)and then calculate the first derivative of equation (10) with respect to *U*, and take the limit of the derivative as 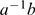 approaches 0 and ψ approaches ∞:
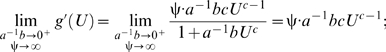
(11)next, calculate the antiderivative (that is, the indefinite integral) of equation (11) with respect to *U*:
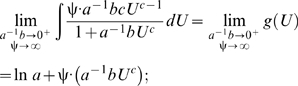
(12)finally, calculate the antilogarithm of equation (12):
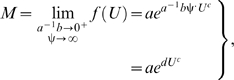
(13)which is equivalent to equation (4).

### Derivation of *f* (*U*) from hydrodynamics

Parameter *c* is inversely related to the swimming efficiency, not the overall energetic efficiency, which takes into account both the power conversion efficiency and the swimming efficiency [6], [34], [35]. If parameter ψ is inversely related to the power conversion efficiency, then the compound parameter *c*ψ is inversely related to the overall energetic efficiency; two dimensionless parameters representing two types of efficiency, like *c* and ψ, can be multiplied, from which the product represents an overall measure of energetic efficiency [6], [34], [35]. Thus, *c*ψ can be derived from the following expression [5]:
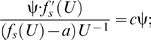
(14)next, rearrange equation (14) such that the first derivative of 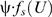, that is, 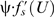, is equal to the first derivative of the natural logarithm of 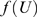, that is, 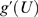, multiplied by 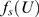:
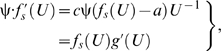
(15)and then rearrange equation (15) such that it equals 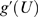:
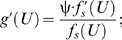
(16)finally, calculate the antilogarithm of the antiderivative of equation (16) with respect to *U*:
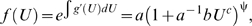
(17)which is conditionally governed by 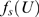.

### The Maclaurin series expansion of *f* (*U*)

Start with equation (3):
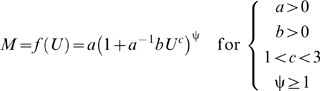
(18)and then transform equation (18) into the composite function:
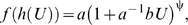
(19)where *h*(*U*) is equal to 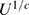; this transformation ensures that the differential function 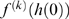 for the Maclaurin series does not equal 0. The series generated by the *k* ( = 1, 2, 3, 4, …, *n*) order derivatives of equation (19) at *U* = 0 is:
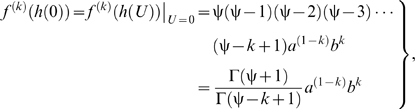
(20)where the gamma function Γ(ψ+1) is equal to ψ! (“ψ factorial”) and is an extension (or a generalization) of the factorials that includes any real number ψ (see equations 4 and 5 in Kleinz and Osler [36]). If ψ is an integer, that is, if ψ equals the floor function 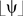, then equation (20) ends after ψ+1 terms because the coefficients on *k* = ψ+1 are zero, and thus *n* must equal ψ. If, however, ψ is not an integer, that is, if ψ does not equal the floor function 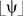, then the series in equation (20) is infinite (*n* = ∞) and converges for all *U* values less than or equal to 
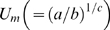
, that is, the maximum sustained *U* at which drag power equals the standard metabolic rate; this is because equation (18) is an adaptation of the binomial series, which converges for any value of 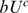 less than or equal to *a* only if ψ does not equal 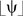 [22]. Incidentally, this convergence has a hydrodynamic and metabolic interpretation worth noting: Weihs [29] stated that drag power (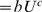: = the propulsive rate of energy) reaches its optimum value (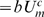) when it equals the standard metabolic rate ( = *a* : = the rate of energy expenditure on internal metabolic processes independent of *U*) (see equation 10 in Weihs [29]). Finally, substituting equation (20) into the Maclaurin series formula results in the series expansion of equation (18):
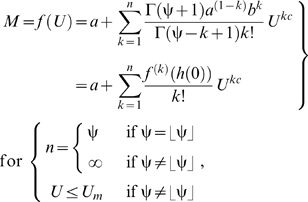
(21)which is equivalent to equation (5).
